# ROS-mediated interplay between brassinosteroids and gibberellic acids antagonistically modulates asymmetric periclinal cell division leading to middle cortex formation in *Arabidopsis* roots

**DOI:** 10.1080/15592324.2025.2577402

**Published:** 2025-11-04

**Authors:** Yoon Kim, Seung Hyun Nam, Soo-Hwan Kim

**Affiliations:** Division of Biological Science and Technology, Yonsei University, Wonju-Si, Republic of Korea

**Keywords:** brassinosteroid, brassinazole resistant 1 (BZR1), gibberellic acid insensitive (GAI), gibberellin, repressor of *ga1-3* (RGA), middle cortex, root development

## Abstract

Asymmetric cell divisions (ACDs) in the root ground tissue of *Arabidopsis thaliana* are essential for middle cortex (MC) formation, which contributes to root architecture and environmental adaptability. Here, we demonstrate that brassinosteroids (BRs) and gibberellins (GAs) antagonistically regulate MC formation via reactive oxygen species (ROS). Brassinolide (BL, a BR) or paclobutrazol (PAC, a GA biosynthesis inhibitor) promoted MC formation and sporadic periclinal cell divisions in root endodermal cell files, whereas brassinazole (BRZ, a BR biosynthesis inhibitor) or GA_3_ suppressed them. Consistently, the BR-signaling gain-of-function mutant *bzr1*-*1D*, the GA-biosynthesis-deficient mutant *ga1*-*3*, and the GA-insensitive mutant *gai*-*1* exhibited elevated H_2_O_2_ levels and increased MC formation. Conversely, the BR-biosynthesis-deficient mutant *det2* and the GA-signaling-enhanced *rga*/*gai* double mutant showed reduced ROS accumulation and MC formation. BL or PAC further enhanced MC-forming effects, while BRZ or GA_3_ diminished them. This antagonistic regulation of BRs and GAs on MC formation was further validated in double mutants: *ga1−3/bzr1-1D* displayed an additive promotion, while *ga1−3/det2* showed a diminished effect on MC formation. The ROS-deficient *rbohD/F* mutant exhibited reduced MC formation and attenuated responses to BL or PAC, and ROS scavenging by potassium iodide suppressed the MC-promoting effects of *bzr1-1D*, *ga1−3*, and *ga1−3/bzr1-1D*. These results identify ROS as a central integrator of BR–GA antagonism, linking hormonal regulation to SHR/SCR-mediated ACDs during MC development in *Arabidopsis* roots.

## Article highlight

• BRs and GAs oppositely modulate middle cortex formation through ROS production, ultimately leading to the antagonistic regulation of asymmetric cell division in the root endodermis.

## Introduction

The root of *Arabidopsis thaliana* serves as a well–established model to investigate how asymmetric cell division (ACD) drives tissue patterning.^1-3^ One notable developmental event is the formation of the middle cortex (MC), a cell layer that arises from secondary asymmetric periclinal divisions in the endodermis during post–embryonic root development, typically occurring between 7 to 14 days after germination (DAG).^4^^,^^5^ As such, the onset of MC formation is commonly used as an indicator of root ground tissue maturation.

Accumulating evidence indicates that tight spatiotemporal regulation of ACDs during MC formation involves a complex interplay among plant hormones, redox signalling, and transcriptional regulators.^4^^,^^6-13^ Among the key regulators are the GRAS family transcription factors SHORT–ROOT (SHR), SCARECROW (SCR), and SCARECROW–LIKE 3 (SCL3), which coordinate ACDs of ground tissues producing cortex, endodermis, and MC.^4^^,^^5^^,^^9^^,^^14-16^ SHR protein, synthesised in the stele, moves to adjacent cell layers where it forms a nuclear complex with SCR and directly activates *CYCLIND6;1* (*CYCD6;1*), promoting ACDs in the cortical–endodermal initials (CEI) and cortical–endodermal initial daughter (CEID) cells to produce the endodermis and cortex.^16-19^ SHR acts in a dose–dependent manner: high levels of SHR suppress MC formation, while intermediate levels promote it.^16^ SCR negatively regulates MC formation by repressing *CYCD6;1* expression, as evidenced by precocious MC development in *scr* and *scr3* mutants.^5^^,^^9^^,^^20^ Indeed, low threshold levels of SHR and SCR are sufficient and act early in the cell cycle to determine the orientation of the division plane, resulting in either formative or proliferative cell division in root stem cells.^21^

In parallel, redox signalling plays a crucial role in ACD regulation.^22-25^ SHR promotes production of hydrogen peroxide (H_2_O_2_) via transcriptional up–regulation of *RESPIRATORY BURST OXIDASE HOMOLOG*s (*RBOH*s), facilitating periclinal cell divisions.^26^ SCL3 contributes to ROS homoeostasis by modulating *PEROXIDASE 34* (*PRX34*) transcription during tissue maturation.^27^

Among plant hormones, gibberellic acid (GA) functions as a molecular clock modulating the timing of asymmetric cell divisions in *Arabidopsis* root ground tissue.^10^ High GA levels suppress MC formation during early root development in a *SHR*-dependent process, whereas later reduction of GA biosynthesis permits MC formation.^5^^,^^8^^,^^9^^,^^14^^,^^28^ DELLA proteins, such as GIBBERELLIC ACID INSENSITIVE (GAI) and REPRESSOR OF *ga1*-*3* (RGA), are negative regulators of GA signalling and play central roles in this process.^29^ In this regard, mutants defective in GA biosynthesis (*ga1−3*) or signalling (*gai−1*) exhibit increased endodermal ACDs and MC formation, highlighting GA’s inhibitory role.^10^ SCL3 modulates GA signalling and contributes to MC repression, and SCL3-DELLA interaction, in conjugation with the SHR–SCR module, plays an important role in the GA–mediated spatiotemporal control of MC formation.^9^^,^^30^

Brassinosteroids (BRs) are plant steroid hormones that play crucial roles in various aspects of plant growth and development.^31^ In roots, BRs regulate growth by modulating BRASSINAZOLE RESISTANT1 (BZR1)/BRI1 EMS SUPPRESSOR1 (BES1)-induced ethylene biosynthesis and by promoting peroxidase–dependent production of superoxide anions (O_2_^−^).^32^ Additionally, the BZR1-/BES1-dependent BR signalling pathway induces ectopic activation of quiescent centre cell division and regulates columella stem cell differentiation in a BR concentration–dependent manner.^33-35^ With regard to ground meristem differentiation, BR–activated BZR1 directly binds to the promoter of *SHR* to induce its expression and physically interact with SHR to enhance the transcription of *RBOH*s, thereby increasing H_2_O_2_ levels.^12^ This redox activation further enhances the SHR–mediated promotion of MC formation. However, it remains unclear how BRs coordinate with other hormonal pathways, particularly with GAs, in regulating MC formation. In this study, we investigate the regulatory crosstalk between BRs, GAs, and ROS during MC formation. We show that BRs and GAs oppositely modulate ROS production, resulting in antagonistic regulation of asymmetric endodermal cell divisions and MC development in *Arabidopsis* roots.

## Materials and methods

### Plant materials and growth conditions

Wild–type *Arabidopsis thaliana* Columbia−0 (Col−0) and *Landsberg erecta* (Ler), BRs– and GAs–related mutants (*bzr1-1D*, *det2*, *ga1−3*, *ga1−3*/*det2*, and *ga1−3*/*bzr1-1D* in the Col−0 background; *gai−1* and *rga/gai* in the Ler background), and ROS–deficient *rbohD/F* mutant were used for phenotypic analysis of MC formation in this study. The *rbohD/F* seeds were kindly provided by Dr. Yuree Lee (Seoul National University, Republic of Korea).

For phenotypic analysis MC formation and H_2_O_2_ production, seedlings germinated on half–strength MS agar medium containing 0.8% phytoagar (pH 5.7) were transferred to fresh 1/2 MS medium supplemented with the chemicals indicated in each experiment. Seedlings at eight days after germination (DAG8) were analysed for MC formation and H_2_O_2_ production. Plants were grown in a growth chamber operating under a cycle of 16 h light and 8 h dark at 23−25°C with 70% humidity. All seedlings were grown vertically.

### Chemicals and treatments

Brassinolide (BL), paclobutrazol (PAC), and potassium iodide (KI) were purchased from Duchefa Biochemie (Netherlands). Brassinazole (BRZ) was kindly provided by Dr. Yoshida (RIKEN, Japan). All other chemicals used in this study were purchased from Sigma–Aldrich (USA), unless otherwise indicated. For chemical treatments, DAG3 seedlings were transferred to 1/2 MS phytoagar plates supplemented with either BL (10^−12^ M), GA_3_ (10^−6^ M), PAC (10^−6^ M), or BRZ (10^−6^ M). For ROS scavenging assays, KI was added to the medium at a final concentration of 1 mM.

### Confocal microscopy and imaging

For phenotypic analysis of MC formation, roots of DAG8 seedlings were stained with propidium iodide (PI, 10 μg/mL) for 1 min and mounted in water. PI signals were obtained using a Zeiss LSM 710 confocal laser–scanning microscope equipped with an argon ion laser (488 nm) and a He/Ne ion laser (543 nm). For observation of PI–stained roots, samples were excited at 543 nm and the PI emission signal was collected at 595−615 nm. MC formation (the frequency of endodermal ACDs) was quantified based on the presence of a clearly distinguishable extra cell layer between the endodermis and cortex. At least 100 roots were analysed for each experiment.

### H_2_O_2_ assays

ROS levels in root tissues were measured using a commercially available Amplex® Red Hydrogen Peroxide Assay Kit (A22188, Invitrogen), according to the manufacturer’s instructions. In brief, roots of DAG8 seedlings grown on a half–strength MS medium in the presence or absence of the indicated chemicals were pulverised in liquid nitrogen. Following the addition of five–fold volumes of 50 mM sodium phosphate buffer (pH 7.4), the samples were thoroughly mixed and incubated on ice for 10 minutes. Subsequently, the mixtures were centrifuged at 12,000 rpm for 20 minutes at 4 °C. The resulting supernatants were collected and used for determination of H₂O₂ concentrations, as previously described.^27^^,^^36^ All experiments were performed at least triplicate, and the data were statistically analysed by Student’s *t*-test using Microsoft Excel (Microsoft, USA).

## Results

### GAs and BRs act antagonistically on asymmetric periclinal cell division, and their respective roles in modulating middle cortex formation are interdependent

Integrated regulation of periclinal cell division by the BZR1-SHR transcriptional module leads to MC formation in *Arabidopsis* roots, while GAs oppositely repress MC formation, suggesting complex regulatory crosstalk between BR and GA in MC development.^5^^,^^8^^,^^12^ Nonetheless, it remains unclear whether and how these hormones interact functionally in the context of MC regulation. To address this gap, we investigated the mutual influence of BRs and GAs on MC formation, focusing on whether these hormones act independently or interdependently through a shared regulatory mechanism.

**Figure 1.**
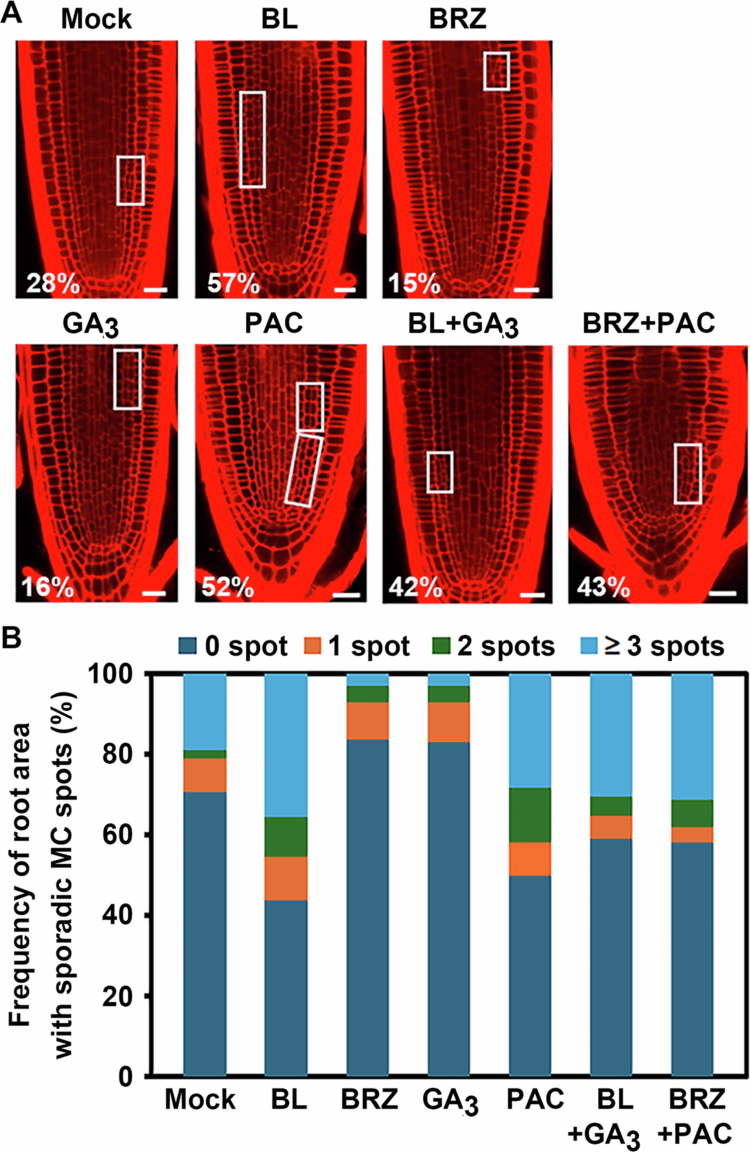
Effects of BR– and GA–related chemicals on MC formation. (A) Representative confocal images of chemical–treated Col−0 roots. Open squares indicate root areas with MC–formed endodermal files. Numbers represent the percentage of plants showing MC formation in roots. Scale bar = 20μm. (B) Frequency of sporadic MC–formed root areas along the endodermal cell files for plants shown in (A). DAG8 seedlings were analysed for MC phenotypes. BL (10^−12^ M), GA_3_ (10^−6^ M), PAC (10^−6^ M), and BRZ (10^−6^ M). *n* > 100 seedlings for each experiment.

First, we tested whether two hormones influence each other in MC formation. To do this, Col−0 seedlings were germinated and transferred to medium supplemented with BR– and/or GA–related compounds, such as BL (a BR), GA_3_ (an active GA), BRZ (a BR biosynthetic inhibitor) or PAC (a GA biosynthetic inhibitor). We found that BL or PAC treatment effectively promoted MC formation: 28% of non–treated Col−0 plants showed asymmetric periclinal cell division in endodermis, whereas 57% of BL–treated and 52% of PAC–treated plants exhibited endodermal cell division resulting in MC formation (Figure 1A). In contrast, BRZ or GA_3_ treatment led to an over 10% reduction in the proportion of MC–formed plants compared with mock–treated Col−0. Next, we investigated interactions between BRs and GAs by co–treating Col−0 seedlings with BL + GA_3_ or BRZ + PAC. Interestingly, BL + GA_3_ co–treatment showed an approximately 15% reduction in MC formation compared to BL treatment alone. A similar reduction was observed for BRZ + PAC compared to PAC alone (Figure 1A). Supporting this inhibitory interaction between BRs and GAs in MC formation, the frequency of MC spot areas with sporadic asymmetric cell divisions along the endodermal cell files showed a similar trend: BL and PAC treatments dramatically increased the MC spot area, whereas BRZ and GA_3_ treatments caused a notable decrease (Figure 1B).

**Figure 2.**
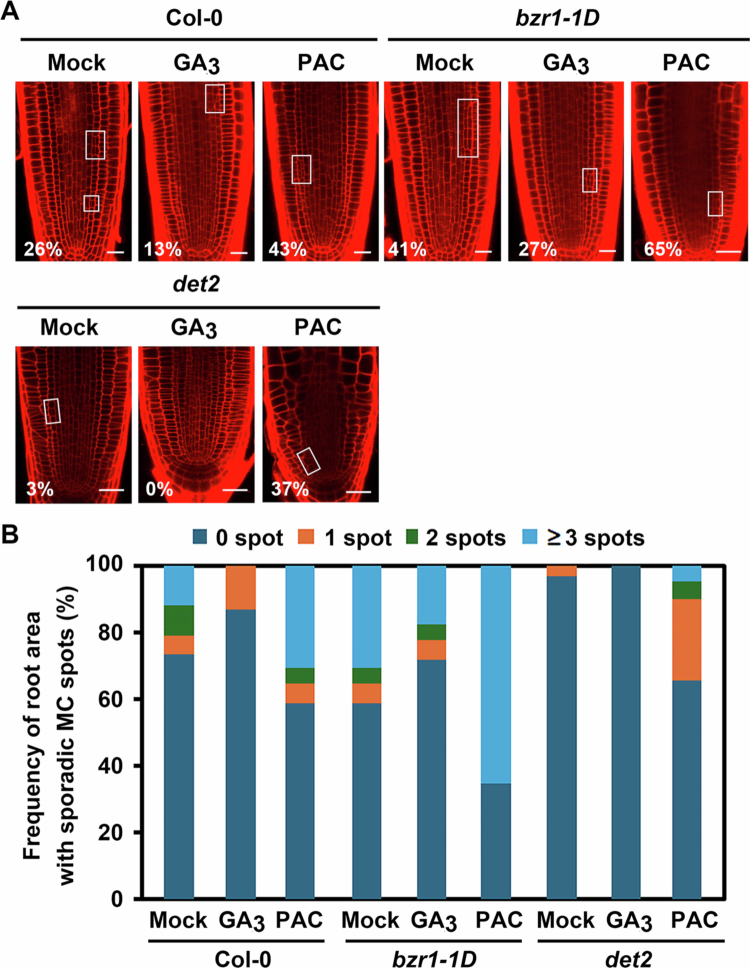
Antagonistic effects of GA–related chemicals on MC formation in BR–biosynthesis and –signalling mutants. (A) Representative confocal images of *bzr1*-*1D* and *det2* roots treated with GA_3_ or PAC. Scale bar = 20μm. (B) Frequency of sporadic MC–formed root areas along the endodermal files for plants shown in (A). Open squares in (A) indicate root areas with MC–formed endodermal files. Numbers represent the percentage of plants showing MC formation in roots. *n* > 100 seedlings for each experiment.

To further explore the functional interaction of BRs and GAs in MC formation, we examined the effects of GA_3_ or PAC on the BR–signalling gain–of–function mutant *bzr1-1D* (in which BZR1 is constitutively active) and the BR biosynthesis mutant *det2* (which exhibits severe BR deficiency). Consistent with the previous report,^12^ *bzr1-1D* exhibited a ~15% increase in MC–formed roots compared to wild–type plants (Col−0), while *det2* showed a prominent decrease (Figure 2A). Supplying GA_3_ to *bzr1-*1*D* seedlings negated the promotion effect on MC formation: the elevated rate in *bzr1-1D* (41%) was reduced to 27% by GA_3_ treatment, whereas PAC treatment of *bzr1-1D* increased the ratio of MC–formed plants to 65%, indicating that GAs can suppress BZR1-mediated, BRs–induced MC formation. Similarly, PAC treatment of *det2* mutants increased the rate of MC–formed roots from 3% (mock–treated *det2*) to 37% (PAC–treated *det2*), suggesting that GA depletion can partially rescue the BR–deficient MC phenotype. Consistently, sporadic endodermal cell divisions were dramatically increased in plants with enhanced BR signalling (*bzr1-1D*), and were further increased in PAC–treated *bzr1-1D* or *det2* plants (Figure 2B). In contrast, GA_3_ treatment on *bzr1-1D* plants efficiently decreased the ectopic MC formation promoted by *bzr1-1D*.

**Figure 3.**
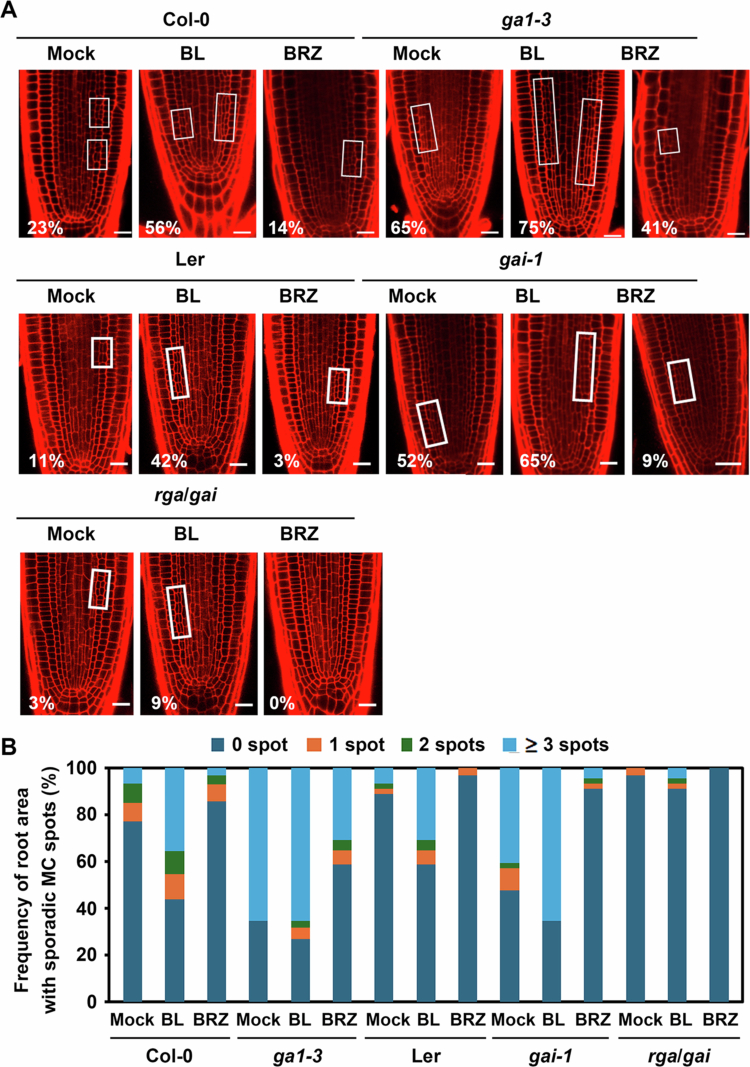
Antagonistic effects of BR–related chemicals on MC formation in GA–biosynthesis and –signalling mutants. (A) Representative confocal images of *ga1−3*, *gai*-*1*, and *rga/gai* roots treated with BL or BRZ. Scale bar = 20μm. (B) Frequency of sporadic MC–formed root areas along the endodermal cell files for plants shown in (A). Open squares in (A) indicate root areas with MC–formed endodermal files. Numbers represent the percentage of plants showing MC formation in roots. *n* > 100 seedlings for each experiment.

Next, we examined the effects of BL and BRZ on the GA–deficient mutant *ga1−3,*^37^ the GA–signalling insensitive *gai−1,*^38^^,^^39^ and *rga−24*/*gai–t6* (*rga*/*gai* in this report),^40^ which show constitutively active GA signalling due to null mutations both in RGA and GAI. As expected, the *ga1*-*3* mutation in Col−0 plants exhibited an enhanced rate of MC formation compared to Col−0 wild type, and BL treatment of *ga1*-*3* further increased the rate from 65% to 75%, while BRZ treatment partially suppressed it from 65% to 41% (Figure 3A). Similarly, *gai−1* plants showed an increased rate of MC formation compared to Ler wild type, and BL treatment further increased the rate from 52% to 65%, while BRZ treatment efficiently suppressed it from 52% to 9%. In contrast, *rga*/*gai* plants, which exhibit constitutively active GA signalling, showed a reduced rate of MC formation (3%) compared to Ler wild type (11%). Moreover, BL treatment of these plants partially increased MC formation to 9%, confirming that GA activity negatively regulates the periclinal endodermal division, while BL acts antagonistically to the GA effect. The same trend of antagonistic regulation between BRs and GAs on ectopic MC formation was observed in BL– or BRZ–treated *ga1*-*3*, *gai*-*1*, and *rga*/*gai*: sporadic endodermal cell divisions were increased in GA–deficient *ga1*-*3* or GA–insensitive *gai*-*1*, and BL treatment further increased the ectopic cell divisions and BRZ treatment produced the opposite effect (Figure 3B).

**Figure 4.**
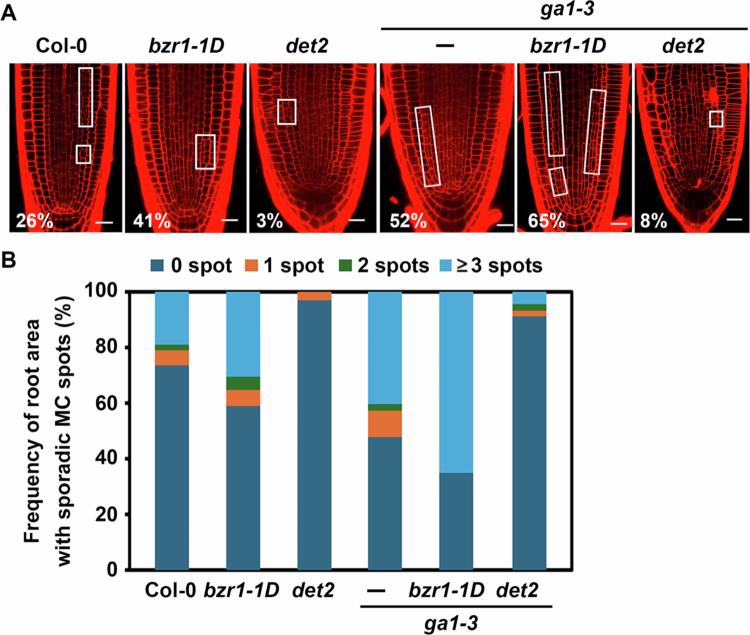
Genetic confirmation of antagonistic regulation of MC formation by BR and GA. (A) Representative confocal images of BR–related, GA–related, and their double mutant roots. Open squares indicate root areas with MC–formed endodermal cell files. Numbers represent the percentage of plants showing MC formation in roots. Scale bar = 20μm. (B) Frequency of sporadic MC–formed root areas along the endodermal files for plants shown in (A). *n* > 100 seedlings for each experiment.

Results presented thus far suggest that BRs and GAs exert opposite effects on the asymmetric endodermal cell division and MC formation. To further assess functional interactions between these two phytohormones, we genetically crossed *bzr1*-*1D* or *det2* with *ga1−3* and examined their MC–related phenotypes. Consistent with observations in Figures 2 and 3, both *bzr1-1D* and *ga1−3* exhibited enhanced MC formation/ectopic periclinal cell divisions along the endodermal files (Figure 4A, B). In contrast, MC formation was dramatically down–regulated in BR–biosynthesis–defective *det2*. We found that the *bzr1-1D*/*ga1−3* double mutant exhibited an approximate 24% increase in MC–formed roots (65%) compared to *bzr1-*1*D* alone (41%) and a 13% increase compared to *ga1−3* alone (52%). This indicates an additive or synergistic enhancement of MC formation due to combined BR signalling activation and GA deficiency. In contrast, crossing *ga1*-*3* with BR–deficient *det2* dramatically negated the MC–promoting effects of *ga1−3*, reducing MC formation from 52% (*ga1*-*3*) to 8% (*ga1*-*3*/*det2*). These results imply that BRs may share signalling components with GAs in modulating asymmetric endodermal cell divisions, highlighting the functional interplay between BR and GA in MC regulation.

### ROS functions as a key integrator of BR and GA signals in regulating their antagonistic periclinal cell division leading to middle cortex formation during ground tissue maturation

It was well documented that ROS and plant hormones, including BRs and GAs, intricately regulate root growth and development, serving as signalling molecules that govern processes such as cell proliferation and differentiation.^23^ BRs have been shown to increase H_2_O_2_ levels in roots while GAs decrease them,^12^^,^^27^ suggesting interplay between BR– and GA–mediated ROS homoeostasis and the resulting antagonistic regulation of MC development.

**Figure 5.**
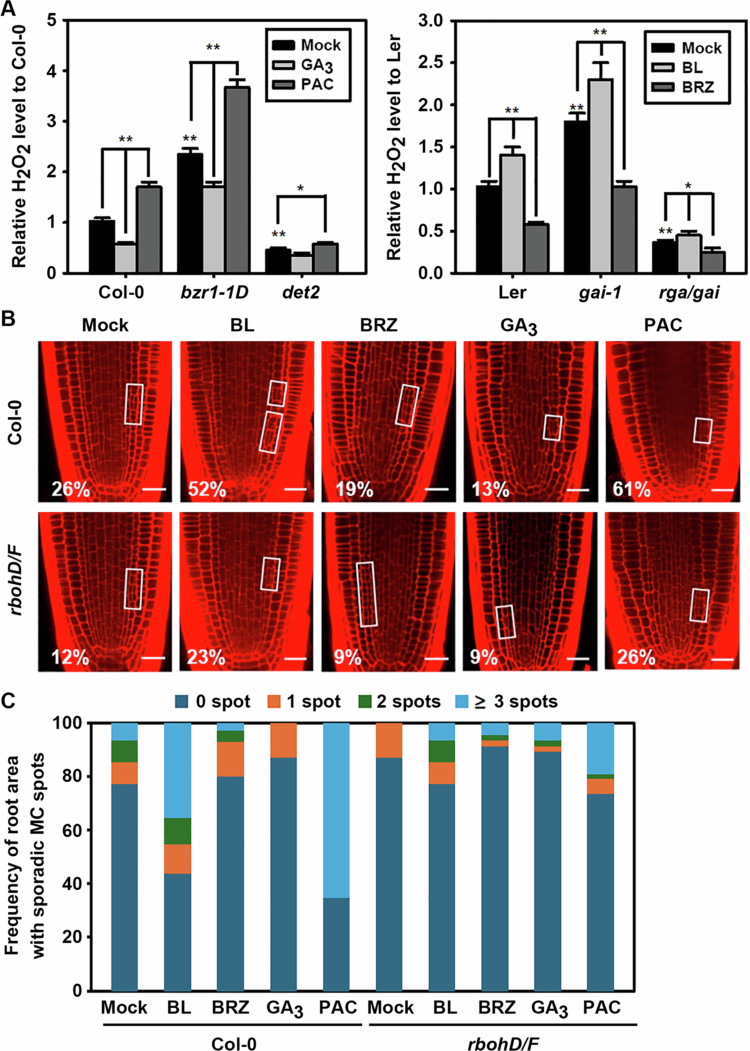
Antagonistic effects of GA– and BR–related chemicals on H_2_O_2_ production and MC formation in BR– or GA–biosynthesis and signalling mutants. (A) Accumulation of H_2_O_2_ in roots of *bzr1*-*1D* and *det2* treated with GA_3_ or PAC, and in roots of *gai*-*1* and *rga*/*gai* treated with BL or BRZ. Bar graphs represent means ± SD relative to mock–treated Col−0 or Ler controls. SD: standard deviation. Statistical differences compared to the mock–treated controls or among bracketed samples are indicated by a single asterisks (*) for *P* < 0.05 and double asterisks (**) for *P* < 0.01. All experiments were performed at least triplicate, and statistical analyses were conducted using Student’s *t*-test in Microsoft Excel. (B, C) Effects of GA– and BR–related chemicals on MC formation in the ROS–deficient *rbohD/F* mutant. (B) Representative confocal images of chemical–treated *rbohD/F* roots. Open squares indicate root areas with MC–formed endodermal cell files. Numbers indicate the percentage of plants showing MC formation. Scale bar = 20 μm. (C) Frequency of sporadic MC–formed root areas along the endodermal files for plants shown in (B). *n* > 100 seedlings for each experiment.

To validate this hypothesis, we comparatively analysed ROS accumulation in the roots of various BR– and GA–related mutants. First, ROS accumulation in roots, determined by measuring H_2_O_2_ levels, was markedly increased in BR–signalling–enhanced *bzr1*-*1D* and decreased in BR–biosynthesis–defective *det2* compared to Col−0 (Figure 5A). Parallel to effects on MC development in *bzr1*-*1D*, GA_3_ treatment of Col−0 or *bzr1*-*1D* plants significantly down–regulated H_2_O_2_ production, while PAC treatment greatly up–regulated ROS production, implying that modulation of GA homoeostasis in roots alters BZR1- and BR–mediated MC development. On other hands, a semi–dominant GA–insensitive mutant *gai*-*1* showed about a 1.8-fold increase in root H_2_O_2_ levels compared to Ler, while *rga/gai* plants, which show constitutively active GA signalling, exhibited a reduction of more than 60% in H_2_O_2_ production (Figure 5A). Conversely, supplying BL to Ler, *gai*-*1*, and *rga*/*gai* plants promoted H_2_O_2_ production, and BRZ treatment reduced ROS production. Collectively, the patterns of ROS accumulation observed in GA_3_- or PAC–treated *bzr1*-*1D* and *det2*, or in BL– or BRZ–treated *gai*-*1* and *rga*/*gai* plants, closely mirrored the antagonistic regulation of MC development, suggesting a strong correlation between ROS levels and the BR– and GA–induced MC development.

SHR induces accumulation of ROS in *Arabidopsis* roots by increasing the *RBOH*s, thereby elevating the levels of H_2_O_2._^12^^,^^26^ To establish a direct causal link between RBOHs–incurred ROS accumulation and BR– and GA–regulated MC development, we examined the effects of BL, BRZ, GA_3_, and PAC on MC formation in the ROS–deficient *rbohD/F* mutant, which lacks RBOHD and RBOHF, two key NADPH oxidases. As reported, the *rbohD/F* mutant exhibited a significant reduction in the proportion of MC–formed roots compared to Col−0 wild type and showed a less frequent sporadic endodermal asymmetric cell divisions (Figure 5B, C). Furthermore, while BL or PAC treatment markedly facilitates MC formation in Col−0 plants (26% for BL–treated and 35% for PAC–treated), this stimulatory effect was substantially reduced in *rbohD/F*, which showed only 9% and 14% increases with BL and PAC treatment, respectively. These results strongly suggests that BRs and GAs antagonistically regulate asymmetric endodermal cell division primarily through their opposing effects on RBOHD– and RBOHF–mediated ROS accumulation in the root.

**Figure 6.**
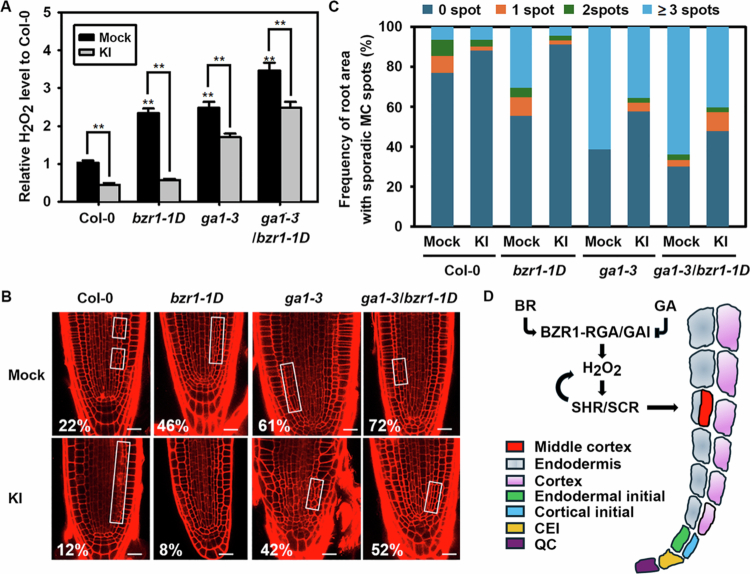
ROS production and MC formation in KI–treated mutants. (A) Accumulation of H_2_O_2_ in roots of *bzr1*-*1D*, *ga1*-*3*, and *ga1*-*3*/*bzr1*-*1D* mutants treated with KI, a ROS scavenger. Bar graphs represent means ± SD relative to mock–treated Col−0 control. SD: standard deviation. Statistical differences compared to mock–treated control or among bracketed samples are indicated by double asterisks (**) for *P* < 0.01. Experiments were performed in triplicate, and statistical analyses were conducted using Student’s *t*-test. (B, C) Effects of KI on MC formation in the mutants shown in (A). (B) Representative confocal images of KI–treated mutant roots. Open squares indicate root regions with MC–formed endodermal cell files. Numbers denote the percentage of plants exhibiting MC formation. Scale bar = 20 μm. (C) Frequency of sporadic MC–formed root areas along the endodermal files for plants shown in (B). *n* > 100 seedlings for each experiment. (D) A schematic model illustrating the ROS–mediated interplay between BR and GA signalling in MC formation. BZR1-activating BR signalling and RGA/GAI–repressing GA signalling oppositely modulate H_2_O_2_ production, ultimately leading to SHR/SCR–mediated antagonistic regulation of asymmetric cell division in the root endodermis. H_2_O_2_ enhances the interaction between BZR1 and SHR, and the resulting BZR1-SHR complex cooperatively promotes ROS production, forming a positive feedback loop that supports MC formation. Arrows represent promotion, while blunted arrows indicate repression.

Next, we accessed the effects of potassium iodide (KI), a ROS scavenger, on ROS– and MC–related phenotypes of Col−0, *bzr1*-*1D*, *ga1*-*3* and *ga1*-*3*/*bzr1*-*1D* double mutants to further validate ROS–mediated interplay of BR– and GA–induced MC formation. As shown in Figure 6, H_2_O_2_ production and the promotion of MC–forming endodermal asymmetric cell divisions were greatly enhanced in *bzr1*-*1D*, *ga1−3* and *ga1−3*/*bzr1*-*1D* plants compared to Col−0 wild type, while supplying KI in the incubation medium greatly reduced these promotive effects, especially in *bzr1-1D* plants (Figure 6A−C). In fact, introduction of the *ga1*-*3* mutation into *bzr1*-*1D* additively increased H_2_O_2_ production and MC formation in the *ga1*-*3*/*bzr1*-*1D* double mutant. These results again confirm that BR– or GA–induced regulation of asymmetric endodermal cell division is tightly correlated with H_2_O_2_ levels, and thus ROS may serve as a converging link for BR– and GA–induced antagonistic MC formation in *Arabidopsis* roots.

## Discussion

The apical region of *Arabidopsis* roots consists of a quiescent centre (QC), which is mitotically inactive, surrounded by various types of stem cell initials, including epidermal–lateral root cap initials, cortical–endodermal initials (CEI), stele initials, and columella initials​​​​​​.^2^^,^^34^ Asymmetric divisions of these initials give rise to distinct root tissues, ranging from the outermost lateral root cap and epidermis to inner ground tissue (cortex and endodermis), pericycle, and vascular tissues, resulting in the radial organisation of the root. The CEI undergoes anticlinal divisions to maintain itself and subsequently produces CEI daughter cells, which divide asymmetrically in a periclinal orientation to generate the inner endodermis and the outer cortex, termed MC.^7^^,^^41^ It is well established that the timing and extent of ACD–driven MC formation are tightly regulated by a network of plant hormones, redox signals, and transcription factors.^3^^,^^7^ In this study, we demonstrated that BRs and GAs exert opposing effects on MC formation in *Arabidopsis* roots, and that these effects converge on the regulation of ROS levels. Our results support a model in which ROS acts as a central integrative signal, mediating downstream transcriptional events that govern asymmetric cell divisions.

BRs and GAs coordinately regulate a wide range of plant growth and developmental processes, including seed germination, pathogen defence, and etiolation responses.^42-46^ Their signalling pathways are intricately linked: for example, DELLA proteins such as RGA and GAI inhibited BZR1-DNA binding ability, while GA–induced and HSP–mediated degradation of DELLAs enhanced BR–induced hypocotyl elongation.^47^^,^^48^ Consistently, *bzr1*-*1D* rescued the short hypocotyl phenotype of the GA–deficient *ga1*-*3* mutant, while ectopic expression of RGA caused destabilization and inactivation of the BZR1.^47^^,^^49^ These reports collectively demonstrate that BZR1-mediated BR signalling in plant growth serves as a positive regulator of the GA pathway, and that RGA/GAI function as negative regulators of the BR pathway. Interestingly, our findings contrast this synergism in growth regulation by revealing antagonistic roles in MC formation. The GA–deficient mutant *ga1*-*3* exhibited enhanced MC formation and elevated H_2_O_2_ levels, and an introduction of the *bzr1-1D* allele into this *ga1−3* (*ga1*-*3*/*bzr1*-*1D*) resulted in an additive increase in ROS accumulation and MC formation, whereas *ga1*-*3*/*det2* double mutants displayed suppressed MC development, suggesting that both BZR1 and RGA/GAI, in contrast to their roles in plant growth, function positively in BRs– and GAs–involved and ROS–mediated MC formation. These results imply that, during root development, BR and GA signalling components may act interdependently through protein–protein interactions, such as those between BZR1 and DELLA proteins,^47^^,^^49^ to regulate ROS homoeostasis and asymmetric division of endodermal cells.

Tissue– and development–specific differential functions of transcription factors (TFs) add multiple layers of regulatory complexity to plant development, highlighting their functional duality in switching between activation and repression modes depending on specific cellular and environmental conditions.^50^ For instance, in stomatal development, BZR1 inhibits meristemoid cell proliferation in cotyledons, whereas in the hypocotyl, it promotes the same developmental process.^51^^,^^52^ The activity of TFs has been reported to be modulated by redox signals, which intricately regulate transcriptional activity by modulating the interaction of redox–responsive TFs with their *cis*-elements, altering their interaction partners, or controlling their subcellular localisation between the cytoplasm and nucleus.^53^ Thus, it is plausible that the BZR1-DELLA complex may enhance BZR1’s transcriptional output by recruiting co–factors or altering chromatin accessibility during ROS–enriched MC formation.

Our findings that ROS accumulation correlates with asymmetric endodermal cell division and MC formation in various BR– and GA–related mutants strongly supports the role of ROS as a key integrative signal in this developmental process. This notion is further reinforced by the marked reduction in MC formation upon treatment with the ROS scavenger KI. Indeed, BR signalling–induced H_2_O_2_ production and the subsequent oxidation of BZR1 enhance its transcriptional activity by promoting its interaction with key regulators of the auxin and light signalling pathways, including AUXIN RESPONSE FACTOR 6 (ARF6) and PHYTOCHROME INTERACTING FACTOR 4 (PIF4), thereby modulating diverse BZR1-mediated processes in plant growth and development.^54^ In addition, H_2_O_2_ significantly increases the interaction between BZR1 and SHR, and the resulting BZR1/SHR complex cooperatively promotes *CYCD6;1* expression to induce periclinal cell division in the root endodermis.^12^ Notably, the BZR1 and SHR/SCL3 module contribute to ROS homoeostasis through the transcriptional regulation of *RBOH*s and *PRX34,*^12^^,^^26^^,^^27^ forming a positive feedback loop that supports MC formation during ground tissue maturation in roots. We suggest that ROS functions not merely as a permissive factor but potentially as an instructive cue in activating the transcriptional programmes governed by SHR and SCR.^12^^,^^55^

In conclusion, we propose a model in which BR and GA signalling pathways converge on ROS biosynthesis to regulate SHR– and SCR–mediated asymmetric cell division during MC development (Figure 6D). This BR–GA–ROS–SHR/SCR axis provides a mechanistic framework for understanding how hormonal and redox cues are integrated to fine–tune developmental patterning in *Arabidopsis* roots. Further studies are needed to elucidate the direct molecular targets of ROS within the SHR/SCR network and to understand how the spatial and temporal regulation of BR and GA signalling contributes to developmental plasticity in the root ground tissue. In particular, studies integrating spatial transcriptomics, chromatin accessibility profiling**,** and protein interaction dynamics in a tissue–specific manner will be crucial for disentangle these complex regulatory networks.

## Data Availability

All data associated with the paper are available in this manuscript. Novel materials used and described in the paper are available for non–commercial research purposes from soohwan@yonsei.ac.kr.
